# 基于微流控技术的细胞水平高通量药物筛选系统的研究进展

**DOI:** 10.3724/SP.J.1123.2020.07014

**Published:** 2021-06-08

**Authors:** Yixiao LIANG, Jianzhang PAN, Qun FANG

**Affiliations:** 浙江大学化学系, 微分析系统研究所, 浙江 杭州 310058; Institute of Microanalytical Systems, Department of Chemistry, Zhejiang University, Hangzhou 310058, China; 浙江大学化学系, 微分析系统研究所, 浙江 杭州 310058; Institute of Microanalytical Systems, Department of Chemistry, Zhejiang University, Hangzhou 310058, China; 浙江大学化学系, 微分析系统研究所, 浙江 杭州 310058; Institute of Microanalytical Systems, Department of Chemistry, Zhejiang University, Hangzhou 310058, China

**Keywords:** 微流控技术, 药物筛选, 高通量筛选, 综述, microfluidic technique, drug screening, high throughput screening, review

## Abstract

药物筛选是新药研发的关键步骤,创新药物的发现需要采用适当的药物作用靶点对大量化合物样品进行筛选。高通量筛选系统能够实现数千个反应同时测试和分析,大大提高了药物筛选的实验规模和效率。其中基于细胞水平的高通量药物筛选系统因为更加接近人体生理条件,成为主要的筛选模式。而目前发展成熟的高通量细胞筛选系统主要基于多孔板,存在细胞培养条件单一、耗时费力、试剂消耗量大等问题,且较难实现复杂的组合药物筛选。微流控技术作为一种在微米尺度通道中操纵和控制微流体的技术,具有微量、高效、高通量和自动化的优点,能较好地克服多孔板筛选系统的不足,为构建细胞高通量药物筛选系统提供了一种高效、可靠的技术手段。微流控系统在细胞培养材料、芯片结构设计和流体控制方面均可灵活变化,能更好地实现对细胞生长微环境的调控和模拟。文章综述了基于微流控技术的细胞水平高通量药物筛选系统的研究进展,按照不同的微流体操控模式,对基于灌注流、液滴和微阵列的3种类型的微流控细胞筛选系统进行了分类介绍,并分别总结了它们的优缺点,最后展望了微流控细胞水平高通量药物筛选系统的发展前景,提出了该领域目前存在的问题以及解决问题的方向。

## Abstract (en)

Drug screening is the process of screening new drugs or leading compounds with biological activity from natural products or synthetic compounds, and it plays an essential role in drug discovery. The discovery of innovative drugs requires the screening of a large number of compounds with appropriate drug targets. With the development of genomics, proteomics, metabolomics, combinatorial chemistry, and other disciplines, the library of drug molecules has been largely expanded, and the number of drug targets is continuously increasing. High-throughput screening systems enable the parallel analysis of thousands of reactions through automated operation, thereby enhancing the experimental scale and efficiency of drug screening. Among them, cell-based high-throughput drug screening has become the main screening mode because it can provide a microenvironment similar to human physiological conditions. However, the current high-throughput screening systems are mainly built based on multiwell plates, which have several disadvantages such as simple cell culture conditions, laborious and time-consuming operation, and high reagent consumption. In addition, it is difficult to achieve complex drug combination screening. Therefore, there is an urgent need for rapid and low-cost drug screening methods to reduce the time and cost of drug development. Microfluidic techniques, which can manipulate and control microfluids in microscale channels, have the advantages of low consumption, high efficiency, high throughput, and automation. It can overcome the shortcomings of screening systems based on multi-well plates and provide an efficient and reliable technical solution for establishing high-throughput cell-based screening systems. Moreover, microfluidic systems can be flexibly changed in terms of cell culture materials, chip structure design, and fluid control methods to enable better control and simulation of cell growth microenvironment. Operations such as cell seeding, culture medium replacement or addition, drug addition and cleaning, and cell staining reagent addition are usually involved in cell-based microfluidic screening systems. These operations are all based on the manipulation of microfluids. This paper reviews the research advances in cell-based microfluidic screening systems using different microfluidic manipulation modes, namely perfusion flow mode, droplet mode, and microarray mode. In addition, the advantages and disadvantages of these systems are summarized. Moreover, the development prospects of high-throughput screening systems based on microfluidic techniques has been looked forward. Furthermore, the current problems in this field and the directions to overcome these problems are discussed.

药物筛选是指从天然产物或者人工合成的化合物中筛选出具有生物活性的新药或者先导化合物^[1]^,是新药研发过程中的关键步骤。创新药物的发现需要采用适当的药物作用靶点对大量化合物样品进行筛选。而随着基因组学、蛋白质组学、代谢组学、组合化学等学科的发展,药物分子库在不断扩大,药物作用靶点也越来越多,这使得药物发现的范围逐渐扩大,药物筛选的工作量急剧增加。因此高通量筛选(high-throughput screening)技术应运而生。高通量筛选系统以分子水平或细胞水平的实验方法为基础,通过自动化操作系统、灵敏快速的检测系统和数据分析系统能够实现数千个反应同时测试和分析,大大提高了药物筛选的实验规模和效率。基于细胞水平的药物筛选系统因为更加接近人体生理条件,实验准确率更高,成为主要的筛选模型。

目前已经发展成熟的细胞高通量筛选系统主要是基于多孔板进行的。它们利用自动化的液体转移分配机械臂完成对液体的多步操控和试剂运输,并使用多孔板扫描仪或者自动化显微镜扫描拍摄技术对细胞的生存情况进行检测。但是这些自动化设备的价格相对较高,不利于普通小型的研究中心或实验室使用。另外,用于药物筛选的生物样品和药物库的成本也较高,而目前常规液体处理设备能够准确操控的液体体积在数百纳升水平。

微流控技术^[2]^是近年来的研究热点之一,其核心是将常规化学、生物等领域所涉及的基本操作单元集成到方寸大小的芯片上,实现对nL级至pL级液体的精准操控,在化学^[3]^、生物学^[4,5]^、医学^[6,7]^等领域都具有广泛的应用。利用微流控技术可以构建高通量、低成本细胞水平的药物筛选系统,系统具有样品及试剂消耗量少、系统高度集成化和自动化等优点。同时,微流控芯片与传统的细胞培养器皿相比,在细胞培养上也具有诸多优势。如通过选择不同的芯片材料或者设计不同的细胞培养腔室结构,更容易实现细胞的三维(3D)培养^[8,9,10]^。因此,基于微流控技术的细胞筛选系统是一种极具潜力的药物筛选系统。

在微流控细胞筛选系统中,通常会涉及细胞的接种、培养液的更换或添加、药物的加入与清洗、细胞染色试剂的添加等操作,这些操作均需通过对微流体的操控得以实现。按照微流体操控模式的不同,可将微流控细胞筛选系统分为3类,分别是灌注流模式、液滴模式和微阵列模式^[11]^。

## 1 基于灌注流模式的微流控细胞筛选系统

基于灌注流模式的微流控细胞筛选系统,通过控制流体连续流过微通道,将细胞接种在微通道中的细胞培养腔室,并完成对细胞的药物刺激。流体的驱动力主要有外部机械泵驱动^[12,13]^、电渗驱动^[14]^、重力驱动^[15]^以及表面张力驱动^[16]^等方式。这类系统的优势在于容易实施,并适用于不同的细胞检测方法,如荧光检测^[17]^、吸光度检测^[18]^、电化学传感器实时检测^[19,20,21,22]^,以及通过质谱对细胞代谢物或其他生物标志物进行检测^[23,24]^。这类系统的局限性主要有:利用微通道进行连续流体输送易导致较高的试剂消耗;当被应用于大规模的药物筛选时,芯片结构通常比较复杂,涉及多种通道、液体控制泵和阀门的设计^[25,26]^。

Wang等^[17]^设计了一种基于灌注流模式的微流控芯片,用于正交化的药物筛选(见图1a)。芯片在水平和垂直两个方向上分别加工有24条通道,通过在芯片中集成气动弹性微阀实现对垂直通道和水平通道的分别控制,完成不同类型的细胞接种和不同药物对不同细胞的正交化刺激。利用该芯片测试了洋地黄皂苷(digitonin)、皂角苷(saponin)、丙烯醛(acrolein)、氯化钴(CoCl_2_)和氯化镍(NiCl_2_)5种物质分别对于小鼠胚胎成纤维细胞(BALB/3T3)、HeLa细胞和牛内皮细胞(bovine endothelial cells)的毒性作用,实现了高密度平行的药物筛选。Park等^[27]^在类似的芯片结构上设计了桥连式的微通道来输送细胞和药物(见图1b),研究了细胞外基质与可溶性转化生长因子-*β*1对二型肺泡上皮细胞(ATII)表型的影响。这种方法不仅可以提高对目标细胞腔室寻址的灵活性,还能减少流体剪切力对细胞的影响。

**图 1:**
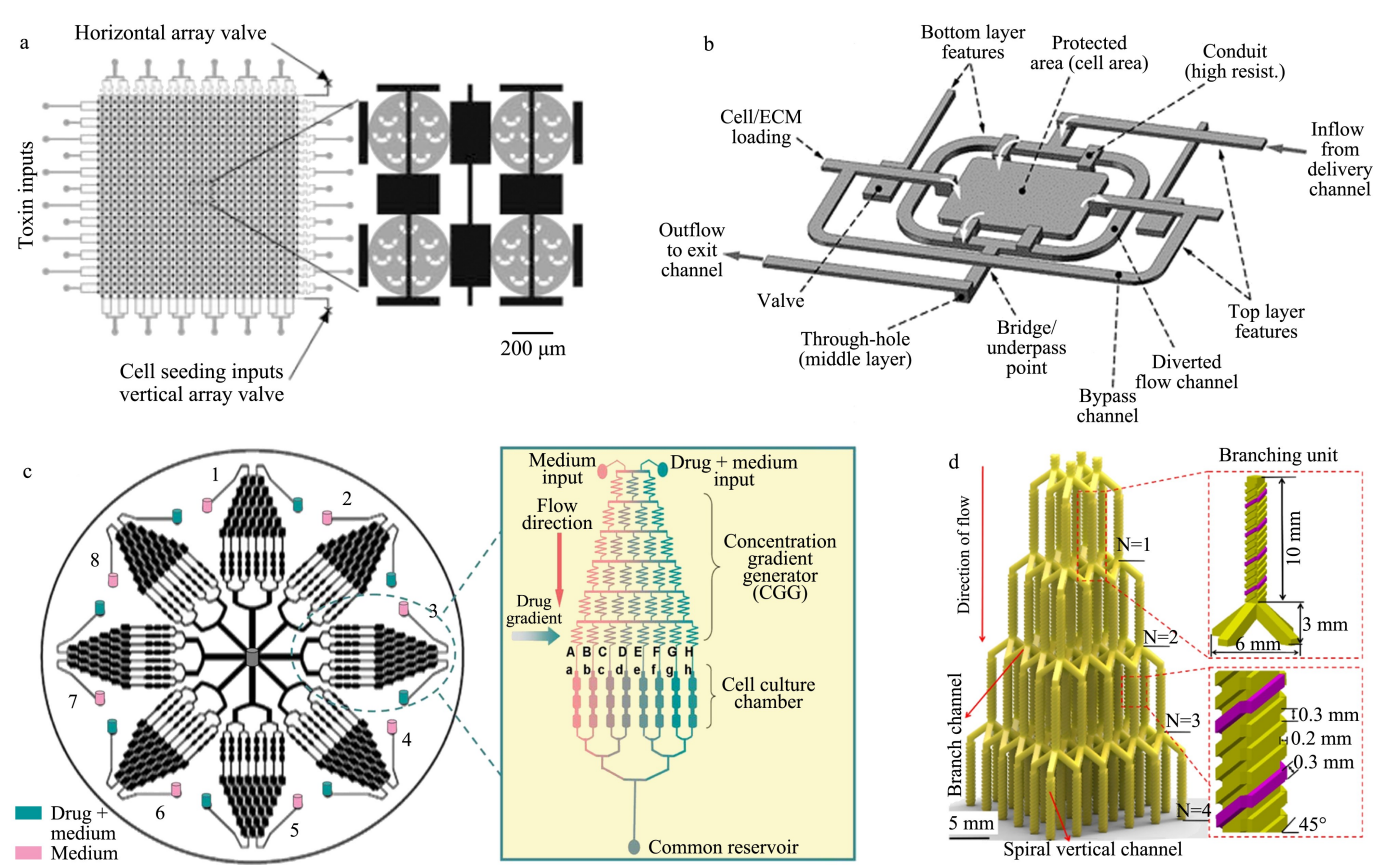
基于灌注流模式的微流控细胞筛选系统

显著的层流效应是灌注流体系的主要特征之一,利用该效应可以在微通道内形成具有较高重现性和稳定性浓度梯度的液流,这类系统被广泛应用于细胞高通量药物筛选中。Ye等^[28]^将浓度梯度药物形成与细胞培养这两个单元集成到一个圆盘芯片中(见图1c)。该芯片含8个具有相同结构的单元,每个单元在上游包含一个“圣诞树”结构的浓度梯度生成器,下游平行连接多个细胞培养腔室,单次可产生64种药物作用条件,实现了对阿霉素(doxorubicin)诱导肝癌细胞(HepG2)凋亡过程的监测。An等^[29]^将两个相似结构的浓度梯度生成器与细胞培养腔室正交连接,评估了姜黄素(curcumin)和肿瘤坏死因子-*α*相关诱导凋亡配体对前列腺癌细胞(PC3)的联合治疗效果。

为了扩大药物浓度梯度范围,Zhang等^[25]^设计了一种非对称流体通道结构的浓度梯度生成器,用于生成具有对数级浓度混合比的药物组合。3D打印技术由于能够制造结构复杂且相互连接的微流体通道,因而被用来加工用于溶液混合的微流控芯片^[30,31,32,33]^。Chen等^[32]^利用3D打印得到了具有三维立体螺旋结构的浓度梯度生成器(见图1d),并测试了4种药物的36种浓度组合对肺癌细胞(A549)的抑制作用。

基于微通道表面的二维(2D)细胞培养模式缺乏细胞生长分化所需的多层次微环境,例如细胞和细胞之间以及细胞和基质之间的相互作用,这可能会导致筛选得到的药物在被应用到临床试验时,治疗效果产生较大偏差^[34]^。3D细胞培养模式因为可以提供更加接近体内条件的微环境^[35,36,37]^,而吸引了越来越多研究人员的关注^[20,38-41]^。如图2a所示,Patra等^[38]^通过对聚二甲基硅氧烷(PDMS)芯片进行预处理,使细胞不能贴壁生长,从而在腔室中形成细胞球并进行药物刺激。该研究组研究了顺铂(cisplatin)、白藜芦醇(resveratrol)和替拉扎明(tirapazamine)3种药物对不同大小的HepG2细胞球的影响。实验结果表明,细胞球的大小和细胞的培养模式(2D和3D)对药物刺激结果均有明显的影响。Toh等^[39]^报道了一种基于3D肝细胞培养的微流控芯片系统(见图2b)。通过在芯片的通道中央加工两排并列的微柱阵列来固定肝细胞,维持肝细胞的3D形态和代谢功能,之后由两侧平行通道引入不同的药物刺激肝细胞,实现对肝细胞的高通量药物毒性测试。Mulholland等^[40]^发展了一种微流控平台,该平台直接利用肿瘤活检中获得的富含癌细胞的多细胞球体进行药物筛选,能够提高筛选结果的准确性。Pandya等^[20]^将电化学传感器集成到微流控细胞筛选系统中,通过叉指微电极实时检测3D凝胶基质中植入的癌细胞对不同药物的敏感性(见图2c)。这些数据可以潜在地预测患者对于不同化疗方法的反应,从而选择出最佳治疗手段,改善癌症治疗效果。

**图 2:**
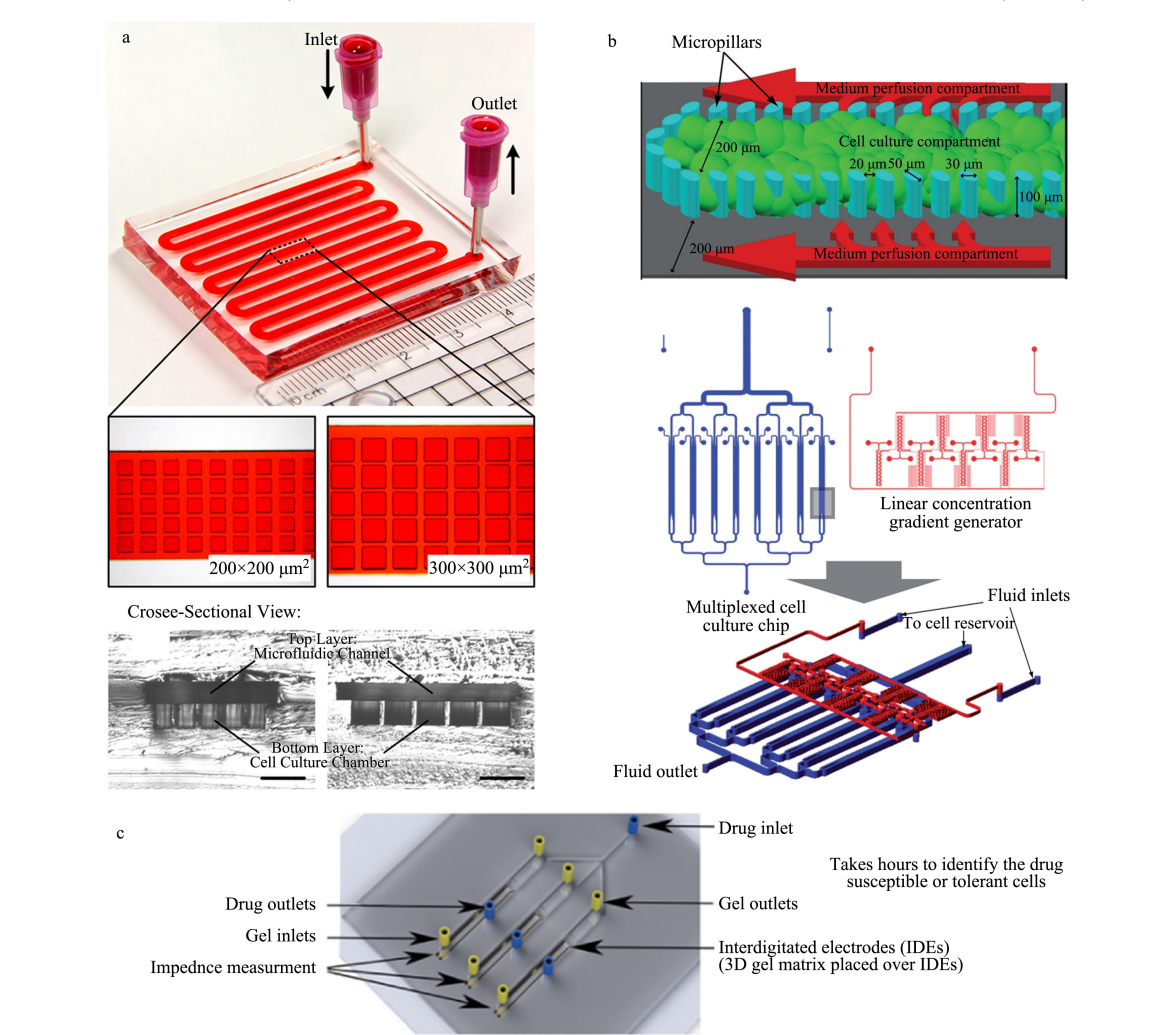
基于3D细胞培养的灌注流模式微流控细胞筛选系统

随着微流控技术的不断发展,多种能够用于模拟人体不同器官及组织间相互作用的器官芯片(organ-on-a-chip)系统已经被报道,包括肺芯片^[42,43,44]^、肾芯片^[45,46]^、脑芯片^[47]^和肠-肝-肿瘤芯片^[48]^等。这些器官芯片系统大部分是基于灌注流模式构建。Huh等^[42,43]^构建了一种具有一定仿生功能的肺芯片(见图3a)。该芯片中,在上下两个PDMS通道之间加工有PDMS多孔膜,PDMS多孔膜上下两侧分别接种肺泡上皮细胞和内皮细胞,肺泡上皮细胞暴露在空气中模拟肺泡的空隙,培养基从下方通道注入模拟毛细血管,通过循环施加真空带动膜的往复运动模拟肺的呼吸。该研究组将白细胞介素-2(interleukin-2)添加到培养基中,再现了其导致肺水肿的药物毒性,并观察到血管生成素-1(angiopoietin-1)以及一种新的香草酸受体4的离子通道抑制剂(GSK2193874)能抑制其药物毒性。Liu等^[49]^设计了一种微通道芯片用于模拟肿瘤诱导的血管生成(见图3b)。芯片包含6个独立的血管生成单元,每个单元由一个开放式的细胞培养腔室和腔室两侧的血管生成通道组成。通过观察内皮细胞在肿瘤细胞诱导下的迁移和血管生成过程,研究了不同的抗血管生成试剂对内皮细胞迁移的抑制作用。Oleaga等^[50]^构建了一种包含心脏、肌肉、神经元和肝脏细胞的多器官芯片(见图3c)。通过重力驱动培养基在不同细胞腔室之间循环流动,实现对细胞的动态培养和药物刺激过程。在芯片上测试了5种药物的细胞毒性,得到的结果与已被公布的动物实验毒性结果基本一致。

**图 3:**
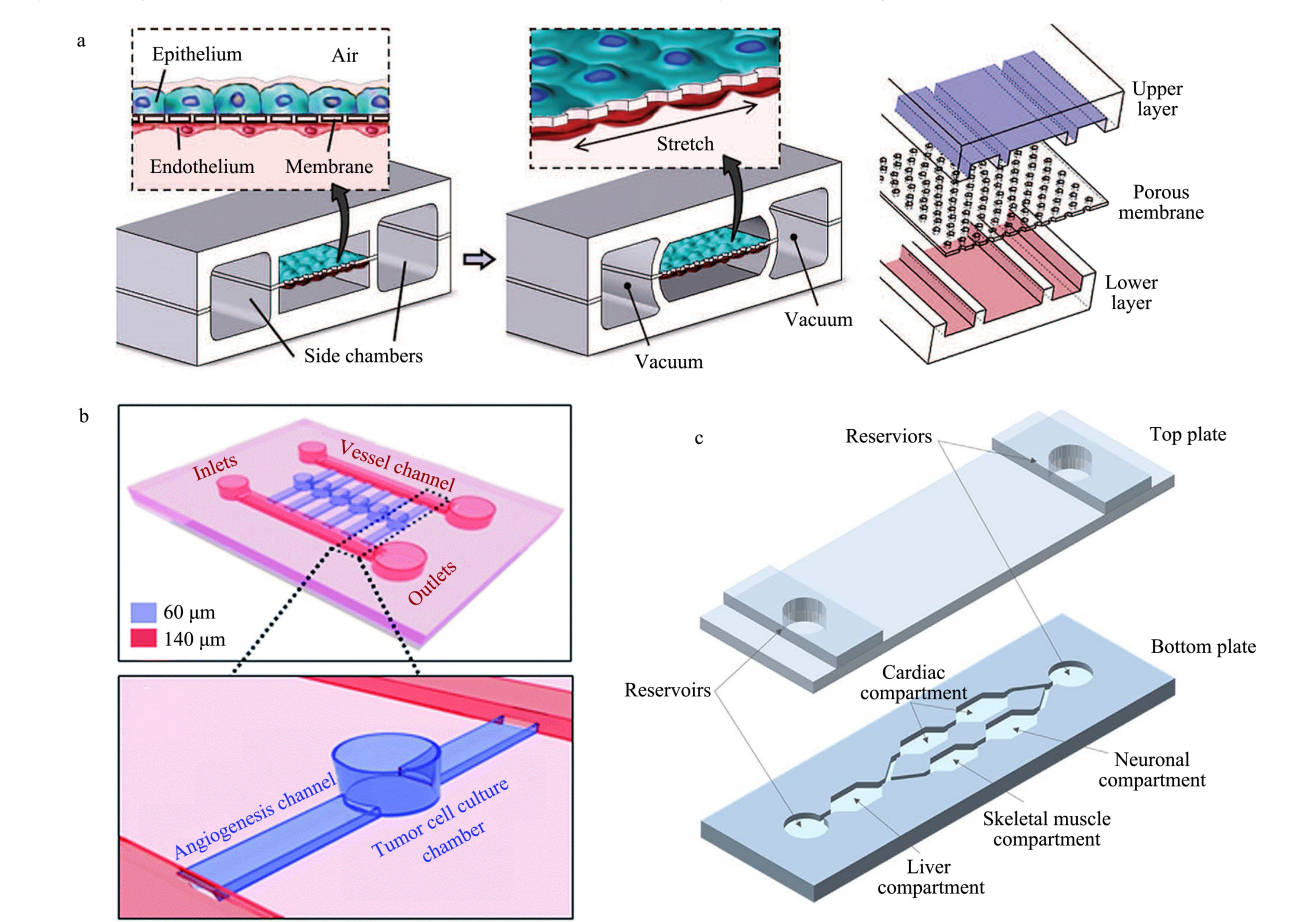
基于灌注流模式的器官芯片系统

## 2 基于液滴模式的微流控细胞筛选系统

自2001年Quake研究组^[51]^提出液滴微流控的概念以来,液滴微流控技术得到了长足发展,在高通量药物筛选领域也得到了广泛应用。基于液滴模式的微流控系统利用互不相溶的两相形成pL至nL级包裹细胞的液滴,在液滴中完成细胞的培养和对细胞的药物刺激等操作^[52]^。这类系统可以在短时间内形成大量细胞液滴,提高实验的通量,并可有效降低试剂的消耗量,但液滴反应器的使用同时也对液滴内营养物质的更新和细胞代谢物的清理提出了挑战。

Clausell-Tormos等^[53]^首次建立了可实现细胞生长的液滴微流控系统,以添加了表面活性剂的氟油(FC40)为连续相(continuous phase),细胞悬浮液为分散相(dispersed phase),生成了FC40包裹的细胞液滴。FC40具有良好的生物兼容性和透气性,因此能够使得细胞在液滴中正常生长。该研究组分别以白血病T细胞(jurkat)和肾细胞(HEK293T)作为悬液细胞和贴壁细胞的代表,证明了即使在液滴体积仅为660 pL的条件下,3 d后细胞的存活率仍能达到80%。

除了产生大小均一的液滴,能够对液滴进行精准操控是实现细胞分析的必要条件。Brouzes等^[54]^构建了一种集成化的液滴微流控系统,用于高通量单细胞药物筛选(见图4a)。首先利用光学编码技术对不同的药物液滴进行标记,形成化合物库,再在电场的作用下将药物液滴和单细胞液滴顺序融合、收集,孵育24 h后将其重新注入微流控芯片中,并将其与含有细胞活性分析试剂的液滴再次融合,对药物刺激结果进行检测分析。Kulesa等^[55]^同样利用电场融合液滴的方法设计了一套适用于组合药物筛选的微流控芯片系统。在该系统中,细胞悬浮液、不同的药物溶液和特异性荧光染料被预先混合并乳化为液滴,这种荧光染料能够分别在不同的激发光下实现对药物的识别和细胞活性的检测。之后将得到的液滴注入PDMS微坑芯片中,由于微坑的独特结构,每个微坑能够同时捕获两个液滴,两个随机被捕获的液滴在电场作用下融合,进行药物对细胞的刺激实验(见图4b)。利用该系统评估了4000多种药物与10种抗生素对大肠杆菌(*Escherichia coli*)的联合作用,成功发现了多种之前未被报道的协同组合。Wong等^[56]^设计了一种PDMS通道芯片,用于形成细胞液滴进行药物筛选(见图4c)。

**图 4:**
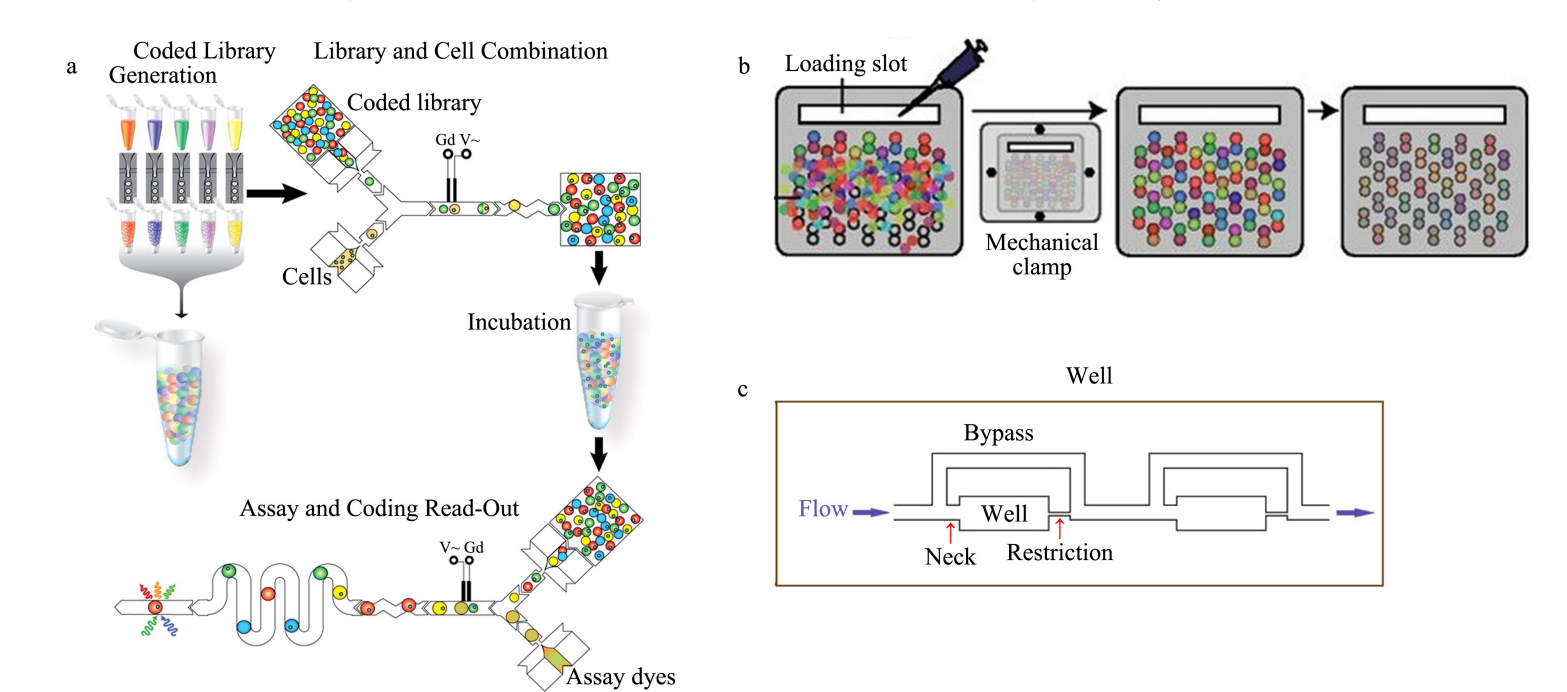
基于液滴模式的微流控细胞筛选系统

每个单通道包含48个细胞培养腔室,当细胞样品从通道入口流入时,由于腔室(well)之后通道(restriction)的限制压力,流体只能进入腔室区域和旁路通道(bypass);随后再通入油相,由于腔室之前通道(neck)的限制压力,油相无法进入腔室,从而截断流体,即可依次在腔室中形成细胞液滴。在该系统中,一个通道中平均截留80000个细胞,用以筛选5种药物作用条件,对于贴壁细胞和悬浮细胞均可适用,可实现在肿瘤切除24 h内,快速、低成本地筛查原发性肿瘤癌细胞。

为了更好地模拟体内肿瘤细胞,目前已经发展了多种基于3D细胞培养的液滴微流控系统^[57,58,59,60,61]^。Wang等^[57]^将HeLa细胞包裹在由藻酸盐和基质胶混合得到的水凝胶液滴中,形成细胞球(见图5a),之后使用长春新碱(vincristine)对细胞球进行刺激。实验结果表明,相比于传统单层培养的细胞,肿瘤细胞球具有更明显的耐药性。Sun等^[58]^开发了一种生成具有核-壳结构的藻酸盐微粒的方法,之后将乳腺癌细胞(MCF-7)和成纤维细胞(human mammary fibroblasts)分别封装在藻酸盐微粒的核与壳中,建立3D共培养的肿瘤模型,模拟体内微小的肿瘤组织(见图5b)。利用该共培养肿瘤球体测定了姜黄素和紫杉醇(paclitaxel)两种抗癌药物的细胞毒性,显示出与常规孔板方法结果相似的肿瘤球耐药性。该方法可快速形成大小一致但组成不同的各种肿瘤球体,应用于大规模抗肿瘤药物的筛选,同时也为细胞的共培养模式提供了新思路。作者所在研究组^[62]^发展了一种在PDMS基片的侧壁上形成细胞球的方法,通过序控液滴阵列(SODA)技术控制毛细管探针在基片侧壁依次形成细胞悬液的悬滴,并稳定地附着在侧壁上,最终培养形成了HepG2细胞球,并完成了阿霉素对细胞球的刺激实验(见图5c)。这种方法的优点在于不仅可以实现细胞的3D培养,还使得悬滴的上方和下方均处于开放的状态,可以方便地完成后续的药物加入、细胞染色以及细胞观察等操作。

**图 5:**
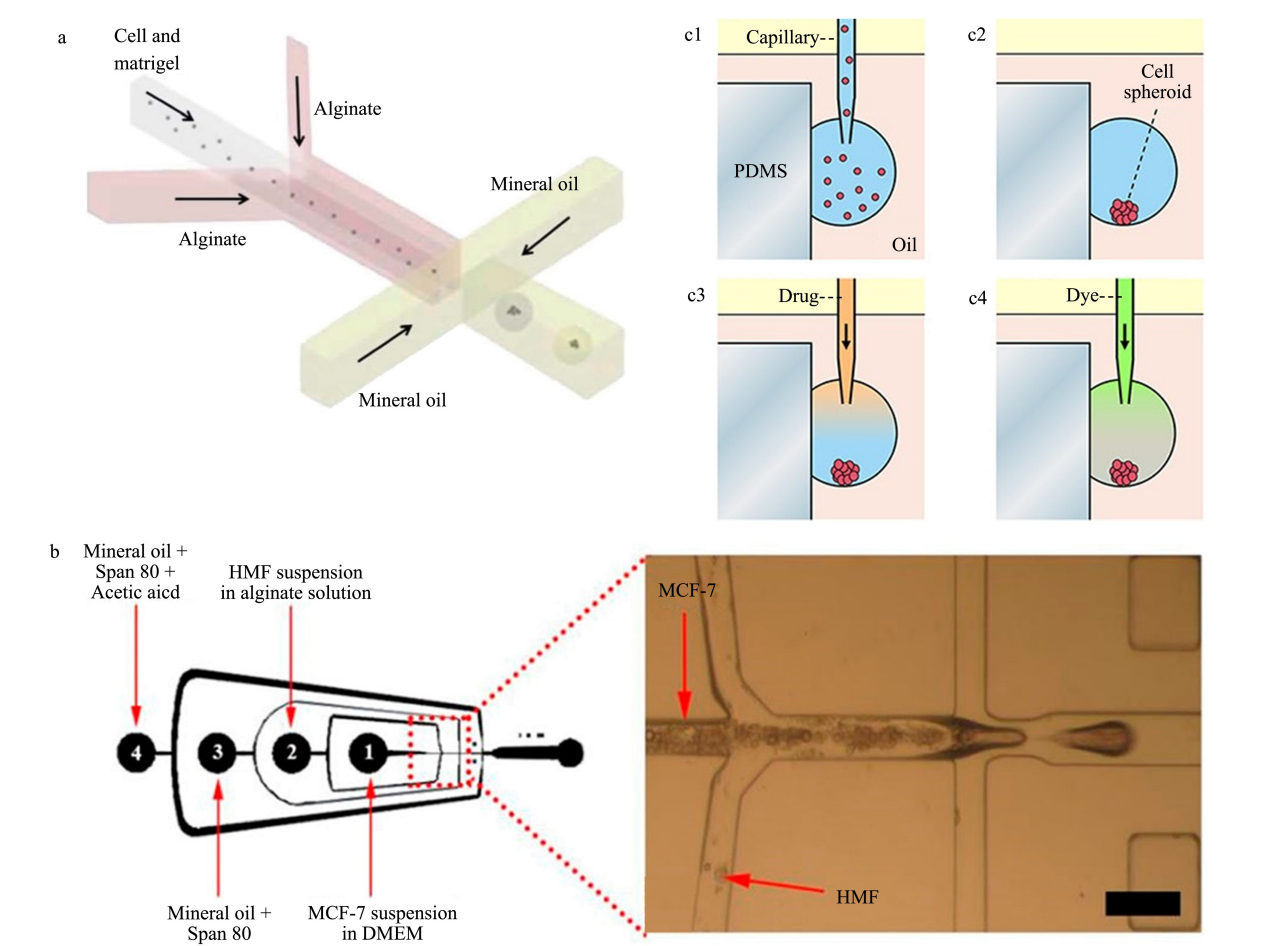
基于3D细胞培养的液滴模式微流控细胞筛选系统

## 3 基于微阵列模式的微流控细胞筛选系统

基于微阵列模式的微流控细胞筛选系统是指在能够适应细胞培养的载体上,通过一定的手段生成细胞液滴阵列,并对细胞液滴阵列进行操控和分析。这类系统能够通过扩大液滴阵列的规模在单次实验中同时筛选大量不同的样品,而且容易实现对细胞液滴阵列的整体操控以提高筛选效率^[63,64,65]^,但同时这些操作的实现也依赖于高精度且配套的液体操控设备。

Lee等^[63]^构建了一种名为水凝胶液滴阵列(DataChip)的微阵列芯片系统(见图6a),并应用于高通量药物筛选实验中。首先利用凝胶打印机在经过处理的玻璃表面生成含有乳腺癌细胞(MCF-7)的水凝胶液滴阵列进行孵育培养,然后将预先制备的药物凝胶阵列(MetaChip)贴合于细胞凝胶阵列上完成药物刺激实验。该系统最多可集成1080个不同条件的液滴阵列,药筛结果与传统的基于96孔板的方法所得到的实验结果相近,但药物消耗量可以降低约2000倍。之后该研究组还提出了通过在微柱阵列芯片的微柱上形成水凝胶细胞液滴用于药物筛选的方法^[66,67]^。

**图 6:**
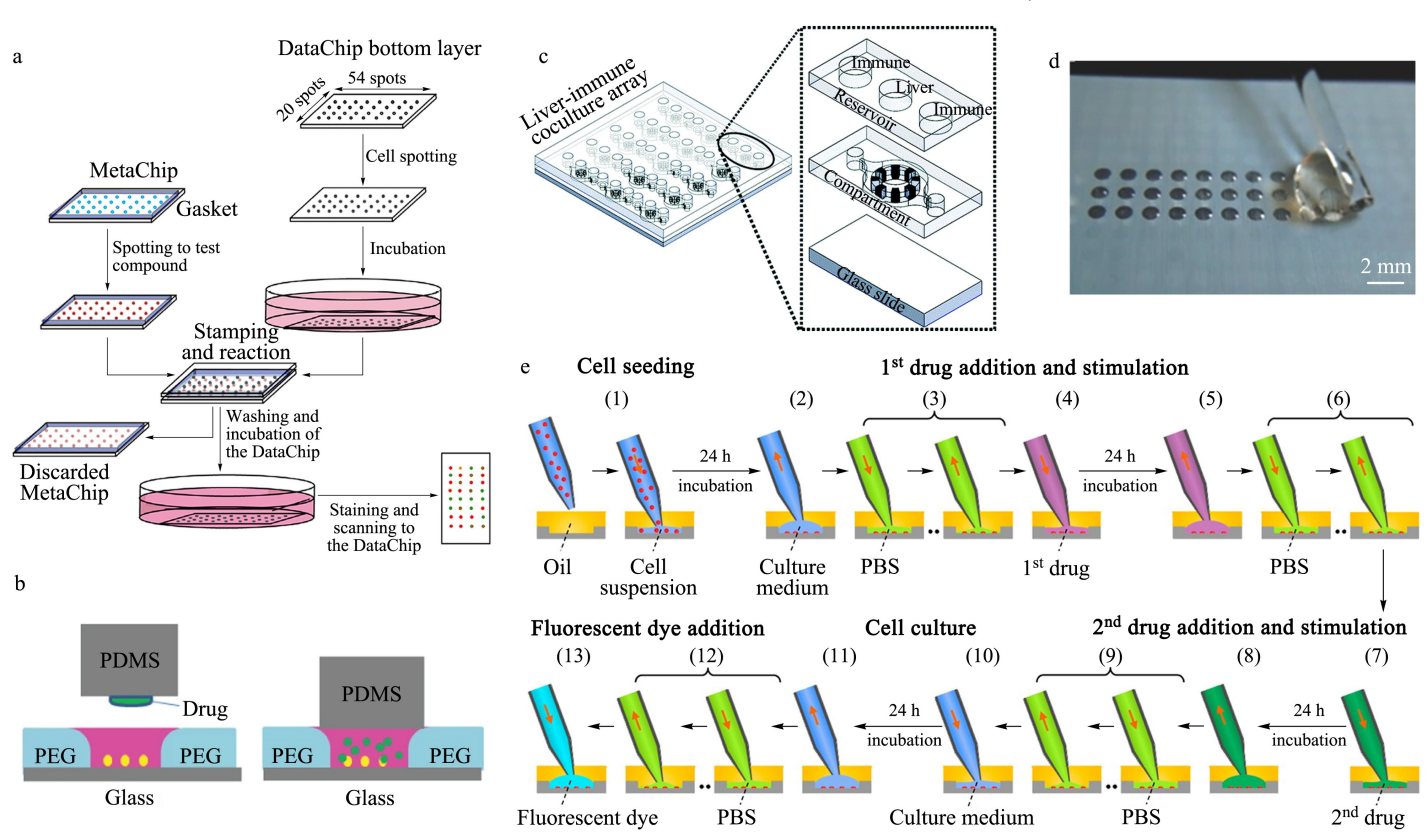
基于微阵列模式的微流控细胞筛选系统

微坑阵列因为易于加工、通量高,在形成细胞液滴阵列上应用非常广泛^[68,69,70]^。Wu等^[68]^利用聚乙二醇(PEG)微坑阵列芯片和PDMS微柱阵列芯片构建了一种夹心结构的微流控细胞筛选系统(见图6b)。先通过点样仪将不同的药物沉积在PDMS微柱阵列上,之后插入接种了细胞的PEG微坑中,实现对细胞的药物刺激。尽管该微坑/微柱阵列芯片仅为普通载玻片大小,但单次可完成2100个测定。利用该系统测试了320种天然化合物对乳腺癌细胞(MCF-7)的化学毒性,还进行了P-糖蛋白(P-glycoprotein)与天然化合物的组合检测,成功筛选出能够增强对MCF-7细胞抑制作用的组合。Li等^[64]^设计了类似的微坑/微柱结构装置。直接以1536孔板提供微坑,制备互补的PDMS微柱,并将其连接到1536孔板的板盖上,加药时翻转板盖对准孔板使微柱插入孔中。通过荧光检测药物与乳腺癌细胞(MDA-MB-231)的相互作用,获得了抑制MDA-MB-231细胞活性的先导单一药物以及具有协同作用的药物组合。Chong等^[71]^设计了一种由同心细胞腔室单元组成的微坑阵列芯片,用于细胞的共培养(见图6c)。每个同心细胞腔室单元由中心腔室和环形腔室组成。肝细胞球体(HepaRG)和U937免疫细胞分别被接种在中心腔室和环形腔室中,经过肝代谢后的活性代谢物通过腔室之间的微通道扩散至邻近的免疫腔室中。通过检测免疫细胞的活化程度,测试了3种药物的致敏性,表明了与传统细胞迁移腔室(Transwell)培养方式相比,该芯片可以更好地区分皮肤过敏的致敏药物和非致敏药物。

利用表面张力来形成细胞液滴阵列也是常用的一种方法。通常会选择对芯片表面进行选择性修饰^[72,73,74,75]^,改变芯片表面特定区域的亲疏水性质,使得细胞悬液被截留在特定区域,从而形成细胞液滴阵列。Popova等^[75,76]^开发了一种形成超疏水-超亲水微图案的方法。利用超疏水和超亲水区域的润湿性差别,自发地形成细胞液滴阵列(见图6d)。通过在这些独立的液滴中进行细胞培养,再加入不同的药物试剂,实现对细胞的高通量分析。Zhang等^[65]^制备了一种由PDMS微坑阵列和超疏水聚合物层组成的超疏水微坑阵列芯片。除了能用来接种细胞液滴,还可以快速实现整个液滴阵列的培养基更换,因此该系统兼容于贴壁细胞和悬浮细胞。

作者所在研究组^[77]^也将SODA技术应用于构建细胞液滴阵列中,发展了一种基于细胞液滴微阵列的药物筛选系统。得益于SODA技术对液体的灵活操控能力(包括液体的量取、吸取、转移、注射、混合等),该系统在一块PDMS芯片上完成了药物筛选过程所需要的全部操作步骤,包括细胞接种、第一个药物刺激、第二个药物刺激、细胞荧光标记等。采用每24 h更换一次细胞液滴培养基的方法,在覆盖了氟油(FC40)的500 nL液滴中完成了长达11 d的细胞培养。该系统被应用于3种抗癌药物黄酮哌啶醇(flavopiridol)、紫杉醇和5-氟尿嘧啶(5-fluorouracil)对A549细胞的药物联用组合筛选中(见图6e),得到了产生最佳抑制效率的药物组合。

## 4 总结与展望

高通量药物筛选是现阶段药物开发的主要途径。微流控技术的出现使生化反应可以在微加工的通道、腔室或nL级甚至是pL级的液滴中进行,这在很大程度上改变了传统高通量筛选系统的工作模式,引起了人们的广泛关注,使得基于微流控技术的细胞筛选系统得到了快速发展,展现出突出的研究潜力和应用价值。

尽管微流控技术在试剂消耗、筛选通量、细胞培养微环境控制等方面与常规方法相比已经展现出明显的优势,但仍然有很大的发展空间。目前文献报道的大部分微流控细胞水平高通量药物筛选系统仍处于实验室研究阶段,尚未达到商品化和通用化的程度。多数系统还难以在超微量和高通量水平下,完成从细胞阵列的形成、培养到加药和检测等全过程操作的自动化。如何自动实现有限细胞的精准量取与分配,如何精准地控制批量微反应器内的细胞培养条件,如何自动实现大规模具有不同组成和浓度药物的加入和刺激,以及如何自动实现筛选结果的快速高通量检测等,是下一步在实现微流控细胞水平高通量药物筛选系统实用化中应该重点解决的问题。此外,目前的微流控系统构建的细胞培养环境与实际体内细胞的微环境还有较大差距。为了能够提高药物筛选结果的准确性,目前研究者们已经不局限于传统独立细胞的培养模型,而是通过构建各种不同结构的器官芯片来进一步模拟人体组织和器官的功能,包括实现细胞的共培养、血液的流动以及物质的传输等,以此来提供一个更加接近人体真实生理和病理条件的研究模型,这也将是今后该领域的重点发展方向之一。

总之,微流控药物筛选系统发展的最终目的是为了减少药物开发过程中对动物实验的需求,甚至是部分替代动物实验,以促进更大规模、更高通量,同时更安全、更有效的药物开发。虽然这还有很长的道路要走,但随着微流控技术的快速发展,微流控细胞水平的高通量药物筛选系统一定会得到进一步完善,实现更为强大和复杂的功能,成为推进全自动药物开发的重要力量。
